# The Effect of Material Parameters of Rubber Asphalt Mortar on Its Friction Performance Under Negative Temperature

**DOI:** 10.3390/ma19030450

**Published:** 2026-01-23

**Authors:** Hui Dou, Bo Li, Peng Zhang, Shengjun Ma, Fucheng Guo, Yan Zhang, Shengan Jiao

**Affiliations:** 1 Gansu Industry Technology Center of Transportation Construction Materials Research and Application, Lanzhou Jiaotong University, Lanzhou 730070, China; 13230040@stu.lzjtu.edu.cn (H.D.); zhangyan1978@lzjtu.edu.cn (Y.Z.); j1739636@163.com (S.J.); 2 Gansu Road & Bridge Construction Group Co., Ltd., Lanzhou 730030, China; 3 School of Civil Engineering, Southwest Jiaotong University, Chengdu 610000, China; 4 Gansu New Development Investment Group Co., Ltd., Lanzhou 730030, China

**Keywords:** rubber asphalt mortar, material parameters, friction performance, friction coefficient, negative temperatures

## Abstract

**Highlights:**

**What are the main findings?**

- Different rubber asphalt mortars present different frictional properties.
- Friction properties are positively correlated with CRC and F/B.
- Friction properties are negatively correlated with filler particle sizes and aging conditions.

**What are the implications of the main findings?**

- Deepen the understanding of the frictional properties of rubberized asphalt mortar.
- Address the research gap regarding the frictional properties of asphalt mortar.
- Promote the development of theories and testing methodologies related to tire-pavement friction.

**Abstract:**

The objective of this study is to investigate the friction performance between tire rubber and rubberized asphalt mortar. The friction force and dynamic friction coefficient (DFC) were selected as the characterization indexes for the friction performance between the tire and the rubberized asphalt mortar, and the tests were carried out using a friction coefficient tester. The variations in material parameters, namely crumb rubber content (CRC), filler-to-binder ratio (F/B), filler particle sizes and aging conditions of rubberized asphalt mortar on friction properties were analyzed, for which significance analysis was carried out. Results show that rubberized asphalt mortar composed of different material parameters exhibit different friction properties. Filler-to-binder ratio and crumb rubber content were identified as significant predictors of the friction properties in rubberized asphalt mortar, and as these factors increase, the friction coefficient between rubber asphalt mortar and tire rubber is increased. Higher crumb rubber content (30%) reduces the temperature sensitivity of friction. In contrast, an increase in F/B exacerbates the temperature-induced variation in DFC, with F/B = 1.2 showing 2.1% DFC increase from −20 °C to −10 °C versus 0.6% for F/B = 0.6. Filler particle sizes, aging degree, and temperature showed no statistically significant effects on DFC (*p* > 0.1).

## 1. Introduction

The safety of vehicles driving on the road surface has always been of utmost importance in the technology of road surface construction [1]. The interaction between rubber tires and road surfaces plays a pivotal role in ensuring vehicle safety and operational efficiency [2]. Adequate friction is essential for maintaining control during acceleration, braking, and cornering, particularly under adverse weather conditions [3,4]. Consequently, understanding the mechanisms governing tire–road friction is crucial for enhancing road safety and vehicle performance [5]. The friction between road surfaces and rubber encompasses two forms, hysteretic friction and adhesive friction [6]. Hysteretic friction is related to the energy dissipation resulting from the damping properties of rubber, which is determined by the characteristics of the rubber material within the tire. Conversely, adhesive friction stems from the shear forces at the tire–road contact interface, especially on smooth, clean, and hard surfaces [7,8].

In the initial operational stage, the material in direct contact with the tire is asphalt mortar that coats the surface of the aggregate. Regrettably, existing research has paid limited attention of the contact between asphalt mortar and tires during this initial operational phase, without taking into account the friction interaction between tire and asphalt mortar, especially for rubberized formulations. Certain scholars have indicated that alterations in the properties of asphalt materials can also exert substantial impact on the friction between road surfaces and tires. Asphalt aging contributes to the enhancement of road skid resistance performance [9,10,11]. Notably, the employment of rubberized asphalt has been demonstrated to remarkably improve skid resistance in comparison with other asphalt materials [12]. This implies that, for mixtures of the same gradation type, the changes are mainly propelled by changes in the properties of the asphalt material. It is of great necessity to study the friction properties of asphalt mortar. This is also in line with the actual operating conditions of road surfaces.

As viscoelastic materials, both rubber tires and asphalt mortar exhibit significant temperature-dependence in their frictional interaction. It is a well-established fact that as the temperature rises, the friction between rubber tires and asphalt pavement decreases [13,14]. Theoretically, this phenomenon can be principally attributed to the fact that elevated temperatures augment the inter-molecular forces within the rubber. This augmentation empowers the tire to disengage from the contact surface and undergo a more rapid recovery process. Consequently, the frictional interaction between the tire and the pavement is mitigated [13,14,15,16,17]. However, this explanation solely takes into account the influence of temperature changes from the rubber tire perspective and fails to address the relationship between the frictional properties of asphalt mortar and temperature variations. In pavement skid-resistance testing, significant shortcomings exist, as relevant standards, such as ASTM E303 [18] and EN 13036-4 [19], only specify testing temperatures typically ranging from +5 °C to +35 °C, entirely overlooking negative temperature conditions. This gap creates notable disparity between the testing temperatures and the actual service temperatures in cold regions during winter, where the pavement temperature often drops well below zero. For instance, in some northern areas, the winter pavement temperature can reach −20 °C or even lower. As a result, the skid-resistance test results obtained under the current standard temperature conditions may not accurately reflect the real-world anti-skid performance of the pavement in these cold-climate scenarios.

Compared with traditional asphalt mixtures, crumb-rubber-modified asphalt mixtures (CRMA)—derived from recycled waste tires—exhibit superior pavement performance and an extended service life [20]. Currently, CRMA is being increasingly applied in highway engineering and is regarded as a promising alternative material for green pavement construction. Furthermore, existing studies have demonstrated that crumb-rubber-modified asphalt pavements also possess outstanding skid resistance [21,22]. However, most relevant research has primarily focused on macro-scale testing of asphalt mixtures, failing to clarify the respective contributions of crumb-rubber-modified asphalt mortar and aggregates to pavement skid resistance Therefore, this study selects rubberized asphalt mortar as the research object. The frictional parameters between rubberized asphalt mortar—composed of varying materials and formulations—and rubber materials are tested and analyzed under negative temperature. This investigation clarifies the influence of material composition and parameter variations in rubberized asphalt mortar on its frictional performance. The findings of this study will contribute to deeper understanding of the frictional properties of rubberized asphalt mortar and address the research gap regarding the frictional properties of asphalt mortar, thereby advancing the theory and testing methodologies related to tire–pavement friction.

## 2. Methodology

**Figure 1:**
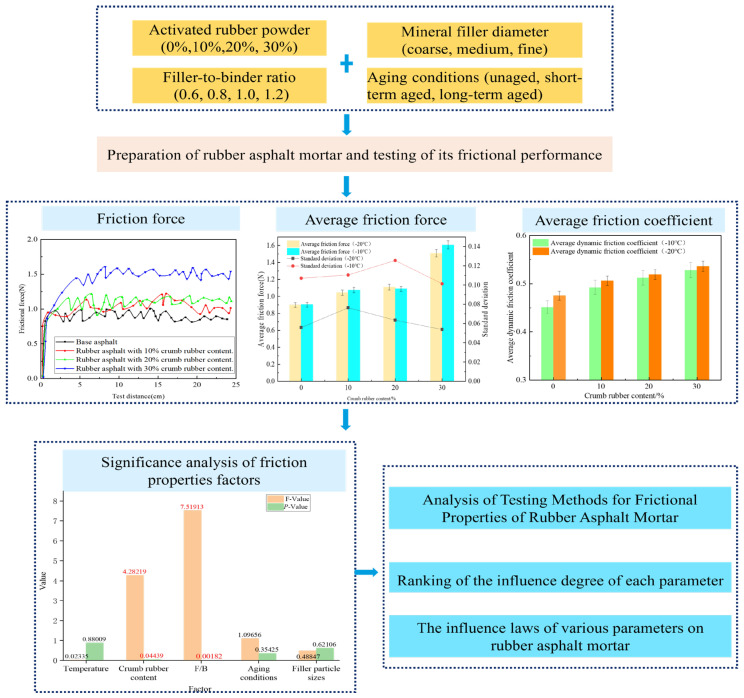
Technical Flowchart.

In this study, a friction coefficient tester was employed to measure the frictional force of rubber asphalt mortar under negative temperature. The experiment considered the following various influencing factors: crumb rubber content (CRC, 0%, 10%, 20%, 30%) [23,24,25], which is recognized as reasonable dosage range in current research; filler particle size (the filler is calcium carbonate, classified into three categories, coarse (0.053~0.075 mm), medium (≤0.075 mm), and fine (≤0.053 mm)); filler-to-binder ratio (F/B, 0.6, 0.8, 1.0, 1.2); and aging conditions (unaged, short-term aged, and long-term aged), for which significance analysis was carried out. The sample parameters are shown in Table 1. The influence of material composition and parameters on the frictional performance of rubberized asphalt mortar under negative temperature conditions was analyzed. The process flowchart is shown in Figure 1.

### 2.1. Materials

#### 2.1.1. Base Asphalt

The base asphalt used was 70# asphalt, which was provided by Gansu Road and Bridge Construction Group Maintenance Technology Co., Ltd., located in Lanzhou, China. Its performance parameters are shown in Table 2, All evaluated technical parameters strictly conform to the specifications stipulated in the Technical Specification for Highway Asphalt Pavement Construction (JTG F40-2004) [26].

#### 2.1.2. Rubber Powder

The rubber powder used in the test was from 40-mesh waste oblique tire rubber powder produced by the normal temperature crushing process of Gansu Wuwei Xin hao yuan Environmental Protection Technology Co., Ltd., located in Wuwei, China. The test results are shown in Table 3. All evaluated performance parameters strictly comply with the specifications stipulated in the Technical Standard for Rubber Asphalt Pavement (CJJ/T 273-2019) [27].

#### 2.1.3. Extraction Oil

In the pretreatment stage of activated rubber powder preparation, the incorporation of extraction oil is essential. The light fractions within the extracted oil facilitate physical dissolution of the rubber powder, inducing structural reorganization of its internal macromolecular chains. This molecular rearrangement enhances the desulfurization and activation efficacy of the rubber powder under high-temperature shear conditions. The chemical composition and physical properties of the extracted oil used in this study are shown in Table 4, which was provided by Gansu Road and Bridge Construction Group Maintenance Technology Co., Ltd.

#### 2.1.4. Asphalt Stabilizer

In powder-modified asphalt systems, for most conventional unmodified inert powders, the dispersed particulate phase within the base asphalt fails to establish a stable crosslinked network with the continuous phase [28]. This phase incompatibility results in reduced modification effectiveness and poor storage stability due to insufficient interfacial interactions. To address this issue, stabilizer is introduced to catalyze branching crosslinking between the active functional groups in the particulate additives and the asphalt mortar, thereby enhancing the thermomechanical performance and homogeneity of the composite materials. The asphalt stabilizer provided by Gansu Road and Bridge Construction Group Maintenance Technology Co., Ltd., with a particle size of 325 mesh. The physical properties of the asphalt stabilizer provided are presented in Table 5, and its main components and corresponding mass fractions are as follows: sulfur accounts for 60%, carbon black accounts for 10%, calcium carbonate accounts for 20%, and rock asphalt accounts for 10%.

#### 2.1.5. Mineral Filler

Limestone mineral filler was selected for this study, which was provided by Gansu Road and Bridge Construction Group Maintenance Technology Co., Ltd., and its test results are shown in Table 6. All evaluated technical parameters strictly conform to the specifications stipulated in the Technical Specification for Highway Asphalt Pavement Construction (JTG F40-2004) [26].

### 2.2. Sample Preparation

#### 2.2.1. Activated Rubber Powder

**Figure 2:**
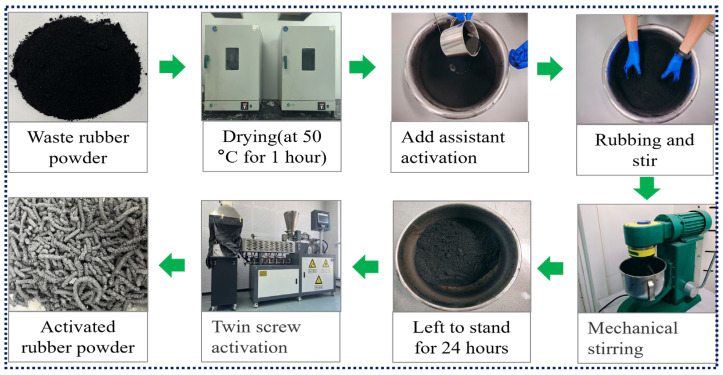
Activated rubber powder preparation process.

The activation of waste rubber powder was performed using a twin-screw extruder (TSE) equipped with five heating modules and one discharging module. The extruder features a screw diameter of 25 mm, length of 1000 mm, and length-to-diameter (L/D) ratio of 40:1. The preparation procedure is as follows: first, the rubber powder is pre-dried in an oven at 50 °C for 1 h. Subsequently, 500 g of the rubber powder is mixed with 50 g (10% of the rubber powder content) of extracted oil and initially stirred until no visible precipitation of the extracted oil is observed. The mixture is then further homogenized using cement mortar mixer for 10 min and left to stand for 24 h prior to activation. For the activation process, the TSE is preheated to 230 °C, after which the pretreated rubber powder is fed into the loading silo. The screw rotation is set to 80 rpm, and the feed mechanism is initiated. The activation of the rubber powder is achieved through the extrusion process, ensuring uniform treatment and enhanced material properties. The preparation process of activated rubber powder is shown in Figure 2.

#### 2.2.2. Rubber Asphalt

We precisely weighed the base asphalt, activated rubber powder, and extracted oil as required by the test protocol. In the initial stage, the activated rubber powder was thoroughly blended with the base asphalt. Then, we agitated this mixture at a temperature of 190 °C and rotational speed of 1000 revolutions per minute (r/min) for a period of 30 min. Subsequently, we carried out shear-grinding operations at 190 °C with shear rate of 6000 revolutions per minute (r/min) over 30 min interval. Finally, we added the extracted oil gradually and stirred the resultant blend at 190 °C and 1000 revolutions per minute (r/min) for 2.5 h to promote the development process.

#### 2.2.3. Rubber Asphalt Mortar

We accurately weighed the rubber asphalt and mineral filler required for the test. We heated the rubber asphalt and maintained it at a temperature of 165 ± 5 °C for 2 h. Subsequently, we stirred the rubber asphalt at a speed of 1000–2000 r/min, gradually adding the mineral filler while continuously stirring. We ensured that the temperature of the blended mortar remained within the range required for the test. After all the mineral filler had been added, we continued stirring for an additional 30 min until no bubbles appeared on the surface of the mixture.

### 2.3. Test Method

#### 2.3.1. Short-Term Aging Test

Short-term aging was simulated via the Rotating Thin-Film Oven Test (RTFOT, JTG E20-2011) [29] to replicate the thermal-oxidative aging process experienced by asphalt binders during storage, transportation, mixing, and paving. The test parameters were strictly controlled as follows: temperature maintained at 163 °C ± 0.5 °C, total test duration of 90 min, sample specification of 35 g ± 0.5 g asphalt binder per sample bottle, and test conditions involving sample bottles mounted on rotating rack (rotational speed: 15 r/min ± 0.2 r/min) with hot air injected at constant flow rate of 4000 mL/min ± 200 mL/min to accelerate oxidative aging. After the completion of aging, the residual samples were collected for subsequent performance testing.

#### 2.3.2. Long-Term Aging Test

Long-term aging was simulated via the Pressure Aging Vessel (PAV, JTG E20-2011) [29] to replicate the long-term oxidative aging process of asphalt binders during pavement service. The test parameters were strictly controlled as follows: temperature maintained at 100 °C ± 0.5 °C, total test duration of 20 h ± 10 min, sample specification of 50 g ± 0.5 g RTFOT-aged asphalt residues (spread into 3.2 mm-thick film), and test conditions involving samples placed in sealed pressure vessel under constant compressed air pressure of 2.1 MPa ± 0.1 MPa throughout the test, with strict control over temperature and pressure stability.

#### 2.3.3. Dynamic Friction Tester (DFT) Method

Currently, the characterization of the frictional performance between tires and the road surface is mainly achieved by testing the anti-skid performance of the road surface. The main characterization indicators are the road surface friction coefficient and the road surface texture depth. The testing methods include the Pendulum Friction Tester (BPT) method and the Dynamic Friction Tester (DFT) method. However, both the BPT and DFT are inevitably affected by the macroscopic texture of the road surface. Therefore, the frictional performance between the asphalt mortar and the tires cannot be directly obtained. To eliminate the influence of pavement texture, this study employed the HP-MXD-01C Friction Coefficient Tester by Jinan Hengpin Electromechanical Technology Co., Ltd. in Jinan, China, to conduct the relevant tests, with the detailed test parameters shown in Table 7.

First, the rubber asphalt mortar, cement board, and preparation device were placed in an oven and heated at 160 °C for 2–3 h to ensure thermal equilibrium. Subsequently, the rubber asphalt mortar was stirred uniformly for 1–2 min with a glass rod. The mortar was then poured onto the cement board, and a 1000 µm preparation device was promptly used to level the surface of the mortar. The thickness of the rubber asphalt mortar specimens was measured using a digital thickness gauge (accuracy ± 0.01 mm) at five different locations (center and four corners) of each specimen. The error range of the five measurement points was controlled within ±0.1 mm. After natural cooling in the ambient environment, the prepared samples were stored for further use. The test plate was placed in an oven or freezer that had reached the experimentally designed temperature and were held at constant temperature for at least 4 h before the test was conducted. One specimen was prepared for each type of rubber asphalt mortar. Three repeated friction tests were conducted on each specimen. The final friction coefficient of the specimen was taken as the average value of the three valid test results.

Then, the rubber asphalt mortar friction specimen was placed face-up and securely fixed on horizontal test bench, ensuring that the specimen was aligned parallel to the longitudinal direction of the bench. A synthetic rubber block, with composition similar to that of commonly used car tires, was selected as the friction pair material. The friction pair material was attached to the sliding rod, aligning it parallel to the direction of the rod’s movement and ensuring that the force measurement system was in an unloaded state.

**Figure 3:**
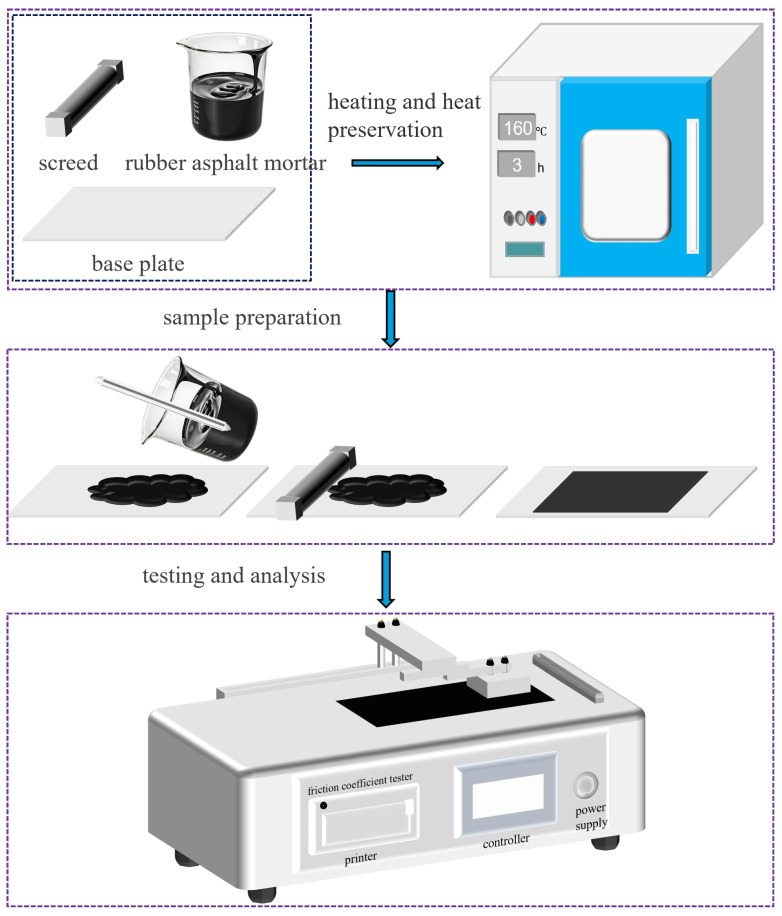
Experimental process.

The test was initiated by clicking the “Start” button. The test automatically concluded once the preset number of test cycles had been completed. For subsequent tests, the “Reset” button was pressed to return the system to standby mode. Upon completion of the test, the instrument interface automatically displayed the friction force–distance curve recorded during the test, as well as the dynamic and static friction coefficients derived from to the maximum friction force. Figure 3 shows the experimental process.

The dynamic friction coefficient (DFC), which characterizes the friction during uniform relative motion between objects, more accurately reflects the motion conditions of the specimen during testing. Therefore, it is adopted to represent the frictional performance of rubber asphalt mortar. However, the instrument can only display the DFC corresponding to the maximum friction force. To obtain the average DFC, this study employs a linear calculation method for derivation. The frictional force is proportional to the DFC and the normal pressure; the relationship can be expressed as Formula (1)

(1) f=μ×N

where *f* represents the frictional force, *μ* represents the DFC, *N* represents the normal pressure.

When testing the same specimen, since the normal pressure applied to the system remains constant, the following relationship can be derived from Formula (2)

(2) $$\frac{f_{average}}{\mu_{average}}=N=\frac{f_{\max}}{\mu_{\max}}$$

where *f_average_* represents the average frictional force, *μ_average_* is the average friction coefficient, *N* is the normal pressure, *f_max_* is the maximum frictional force, and *μ_max_* is the maximum DFC.

Here, the maximum DFC is automatically displayed by the equipment after the test, while the maximum frictional force and the average frictional force can be derived from the frictional force curve. Therefore, the average DFC can be calculated using Formula (2). This derivation is based on the approximate assumption that the normal force N remains strictly constant.

## 3. Results and Discussion

### 3.1. Influence of Crumb Rubber Content on the Frictional Properties of Rubber Asphalt Mortar

**Figure 4:**
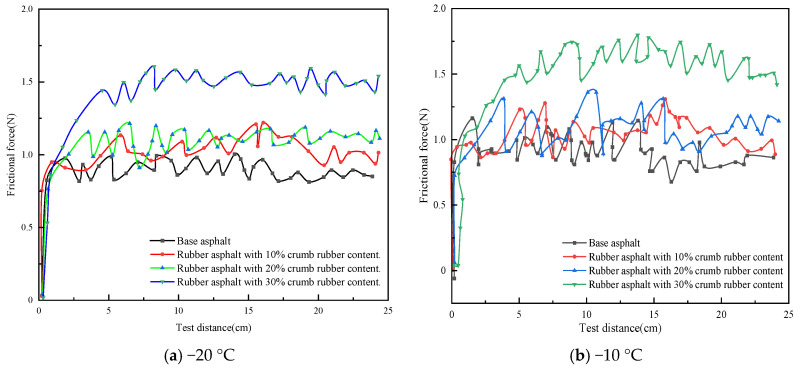
The relationship curves between frictional force and distance for rubber asphalt mortar with different crumb rubber contents at −20 °C and −10 °C.

The relationship curves between frictional force and distance for rubber asphalt mortar with different crumb rubber contents at −20 °C and −10 °C are illustrated in Figure 4. The rubber asphalt mortar was prepared with a filler-to-binder (F/B) ratio of 1.0 using unaged asphalt, and the particle size of the mineral powder was medium.

As shown in Figure 4, the rubber asphalt mortar with different crumb rubber contents at both temperatures exhibits relatively stable nonlinear vibration behavior. As depicted in Figure 4a, it can be observed that at −20 °C, the friction force curve of the base asphalt mortar without crumb rubber is the lowest. In contrast, the curves for rubber asphalt mortar with 10% and 20% crumb rubber content lie in the middle, and the curve for 30% crumb rubber content is the highest. As depicted in Figure 4b, it can be seen that the friction force curves of rubber asphalt mortar at −10 °C follow a similar trend. However, the difference lies in the amplitude of fluctuations; at −20 °C, the fluctuations in the frictional force curves of rubber asphalt mortar are relatively small, whereas at −10 °C, the fluctuations are more pronounced.

**Figure 5:**
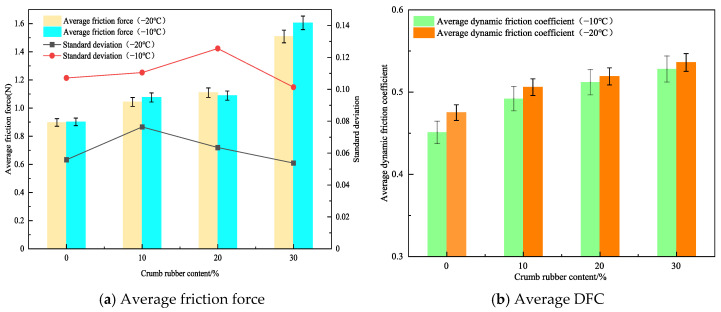
Average friction force and DFC of rubberized asphalt mortar with different crumb rubber contents.

To quantitatively analyze the variation characteristics of the friction force curves of rubber asphalt, the average frictional force and the average DFC at both temperatures were calculated for analysis. The standard deviation was used to characterize the dispersion of the data in the curves. To eliminate systematic errors in the initial stage of the test, data points after test distance of 5 cm were used for statistical analysis. The results of the average friction force and the DFC are shown in Figure 5.

According to Figure 5, increasing rubber powder content (0–30 wt%) significantly improved both friction force and DFC. At −20 °C, the average friction forces of rubber asphalt mortar with 10%, 20%, and 30% rubber powder content are 16.2%, 23.6%, and 68.1% higher, respectively, than those of the base asphalt without rubber powder. At −10 °C, the average friction forces are 19.2%, 20.6%, and 78.0% higher, respectively, than those of the base asphalt without rubber powder. This indicates that the higher the rubber powder content, the greater the friction force. This is primarily due to the following four reasons: first, when rubber powder is added to base asphalt, it modifies the asphalt’s microstructure [30]. As the rubber powder content rises, rubber particles form a dispersed-phase structure. These particles, with their rough and irregular shapes, enhance mechanical interlocking when in contact with the friction pair material; second, with more rubber powder, the number of rubber particles in the rubberized asphalt mortar grows, distributing within the asphalt and enlarging the contact area with the friction pair material [31]; third, adding rubber powder increases asphalt viscosity [32]. Higher viscosity strengthens the cohesion between mortar molecules. When the friction pair material contacts the rubberized asphalt mortar, greater intermolecular forces must be overcome for relative sliding, increasing adhesive friction; finally, rubber’s excellent elastic and damping properties allow rubber particles in the friction process to absorb and dissipate energy [33], impeding relative movement between the friction pair and increasing frictional force. As rubber powder content increases, this energy-dissipation effect becomes more pronounced.

Furthermore, by analyzing the standard deviation values at different temperatures, it is observed that the standard deviation values at −20 °C generally range between 0.05 and 0.08, while those at −10 °C range between 0.10 and 0.13. This further indicates that with decreasing temperature in the negative temperature range, the fluctuation amplitude of the mortar’s surface friction force is slightly reduced. This phenomenon may be attributed to the fact that as the temperature increases, the rubberized asphalt mortar gradually transitions from a glassy state to a highly elastic state. During this transition, the internal molecular interactions within the rubberized asphalt mortar strengthen, causing the entire system to become relatively unstable, which in turn leads to greater fluctuations in the curve.

From Figure 5b, it can be observed that at −20 °C, the average DFC of rubber asphalt mortar with 10%, 20%, and 30% rubber powder content are 3.1%, 4.4%, and 6.1% higher, respectively, than those of the base asphalt mortar without rubber powder. At −10 °C, the average DFC of rubber asphalt mortar with different rubber powder contents are 2.4%, 5.5%, and 6.8% higher, respectively, than those of the base asphalt mortar. Higher rubber content (30%) decreased temperature susceptibility, evidenced by narrowed DFC variations between −20 °C and −10 °C (ΔDFC = 0.8% versus 1.4% for 10% content). Macroscopically, this indicates that the average DFC of rubber asphalt mortar increases with the increase in rubber powder content. Additionally, by comparing the average DFC at different temperatures, it is found that the average DFCs at −10 °C are higher than those at −20 °C, with increases of 5.3%, 2.8%, 1.4%, and 1.5%, respectively. This may be attributed to the fact that as the crumb rubber content increases, the temperature sensitivity of rubberized asphalt mortar decreases accordingly under negative temperature conditions [34,35]. This implies that the performance variation in the rubberized asphalt mortar under different temperature conditions becomes relatively smaller, enabling it to provide higher friction more stably without significant performance fluctuations due to temperature changes.

### 3.2. Influence of Mineral Powder Particle Size on the Frictional Properties of Rubber Asphalt Mortar

**Figure 6:**
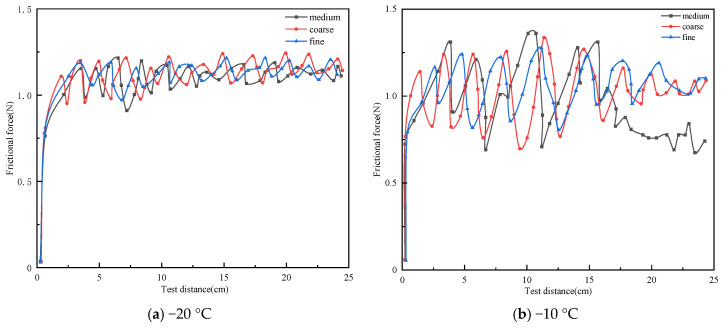
The relationship curves between frictional force and distance for rubber asphalt mortar with different mineral filler particle sizes.

The mineral powder was categorized into the following three types based on particle size: coarse (0.053–0.075 mm), medium (0–0.075 mm), and fine (0–0.053 mm), to prepare rubber asphalt mortar. The F/B was uniformly set at 1.0, and crumb rubber content accounted for 20% of the total mass of the rubber asphalt mortar. The friction force–distance curves of the rubber asphalt mortar with different mineral powder particle sizes at −20 °C and −10 °C are illustrated in Figure 6.

**Figure 7:**
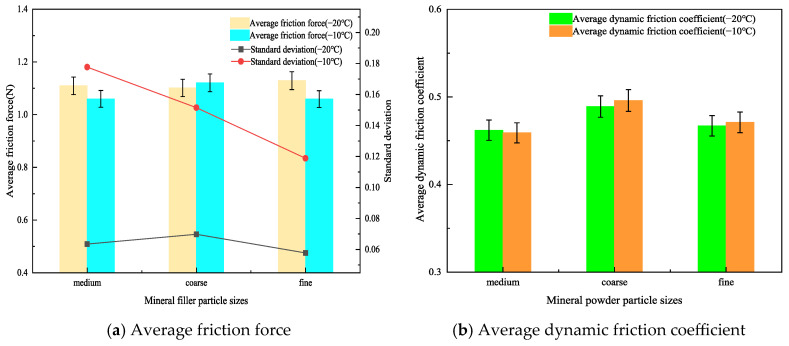
Average friction force and DFC of rubberized asphalt mortar with different mineral powder particle sizes.

From Figure 6a, it is observed that at −20 °C, the friction force curve of the rubber asphalt mortar fluctuates within range of 0.8 to 1.2 N, and the overall shapes of the friction force curves for different mineral powder particle sizes are similar. From Figure 6b, it is found that at −10 °C, the fluctuation range of the curves is between 0.7 and 1.3 N, with the friction force roughly oscillating around 1.0 N. This indicates that the fluctuation of the friction force curves at −10 °C is more pronounced compared to that at −20 °C. The average frictional force and DFC of the rubber asphalt mortar under different mineral powder particle sizes were calculated, as shown in Figure 7.

From Figure 7a, it can be observed that at −20 °C, the average frictional forces for coarse, medium, and fine mineral filler particle sizes are 1.10 N, 1.11 N, and 1.13 N, respectively. At −10 °C, the average frictional forces are 1.12 N, 1.06 N, and 1.06 N, respectively. This indicates that under negative temperature and conditions, the contribution of different mineral filler particle sizes to the frictional performance of rubber asphalt mortar is relatively small, which was also affected by the extremely narrow particle size ranges selected in the study. Changes in the mineral filler particle sizes would inevitably lead to variations in the contact area between friction pair material and rubber asphalt mortar, resulting in viscous energy dissipation and consequently causing significant differences in the frictional force between friction pair material and rubber asphalt mortar.

Additionally, by comparing the standard deviation values at different temperatures, it is observed that at −10 °C, the standard deviations for medium, coarse, and fine particle sizes are 0.18, 0.15, and 0.12, respectively. In contrast, at −20 °C, the standard deviation remains relatively stable at around 0.06. This indicates that under negative temperature conditions, as the temperature decreases, the fluctuation amplitude of the friction force curve gradually diminishes. This trend exhibits similar characteristics to the standard deviation of the friction force in rubber asphalt mortar under different crumb rubber contents, and it is speculated that the possible reason for this phenomenon is related to the glass transition temperature of rubberized asphalt.

From Figure 7b, it can be observed that at −20 °C, the rubber asphalt mortar prepared with coarse mineral powder exhibits higher average DFC, while the average DFC of the mortar prepared with fine and medium mineral fillers is nearly identical. This phenomenon may be attributed to the fact that, under the same sample preparation process, the particle size of the coarse mineral powder is larger than that of the medium and fine powder, which enhances the mechanical interlocking capability of the rough peaks on the mortar surface. As a result, the rubber asphalt mortar with coarse powder demonstrates higher average DFC. Additionally, for the same particle size, the average DFC at −10 °C does not show a significant difference compared to that at −20 °C, indicating that under negative temperature conditions, the influence of mineral powder particle size on the frictional performance of rubber asphalt mortar is relatively minor. The selection of an excessively narrow particle size range for mineral filler maybe affected the accuracy of the results and it is necessary to further expand the particle size range for supplementary tests in subsequent research.

### 3.3. Influence of Filler Binder Ratio on the Frictional Properties of Rubber Asphalt Mortar

**Figure 8:**
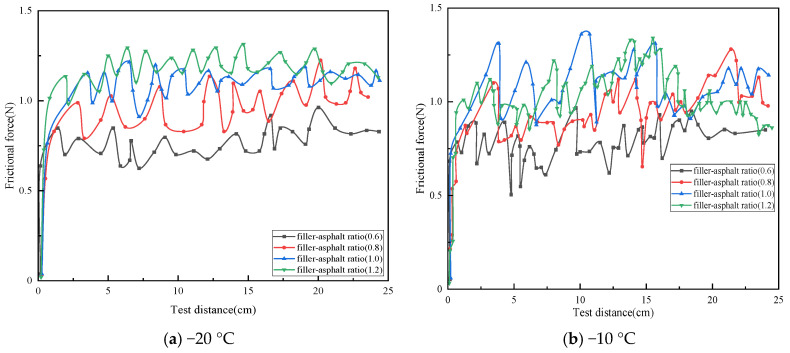
The relationship curves between frictional force and distance for rubber asphalt mortar with different F/B.

The relationship between the frictional force and the test distance for rubber asphalt mortar prepared with medium particle size mineral filler, 20 wt% crumb rubber, and filler-to-bitumen ratios (F/B) of 0.6, 0.8, 1.0, and 1.2 is presented in Figure 8.

**Figure 9:**
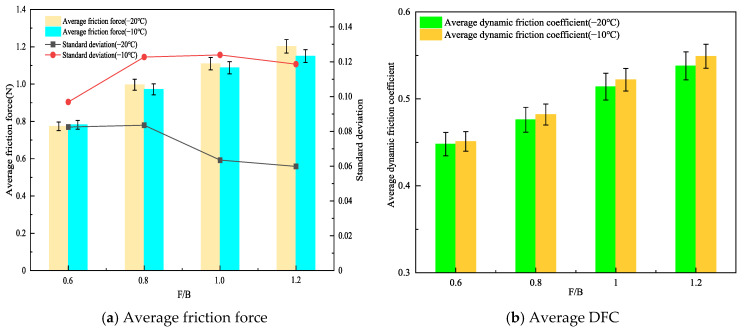
Average friction force and DFC of rubberized asphalt mortar with different F/B.

As clearly shown in Figure 8, at −20 °C, the friction curves gradually shift upward with increasing filler-to-bitumen ratio (F/B), and the fluctuation amplitude of the friction force remains within approximately 0.3 N. In contrast, at −10 °C, the amplitude of the friction force curves increases, and the curve patterns lack discernible regularity. To gain deeper insights into the results, the average friction force and dynamic friction coefficient (DFC) of the friction curves for rubber asphalt mortar prepared with different F/B ratios were computed, as presented in Figure 9.

As shown in Figure 9a, at −20 °C, the average friction of rubber asphalt mortar with F/B of 0.8, 1.0, and 1.2 increased by 28.8%, 43.4%, and 55.4%, respectively, compared to that with F/B of 0.6. At −10 °C, the average friction of rubber asphalt mortar with F/B of 0.8, 1.0, and 1.2 increased by 24.3%, 39.1%, and 42.0%, respectively, compared to that with F/B of 0.6. These results indicate that the average friction of rubber asphalt mortar increases with the rise in the F/B. This is primarily due to two reasons; first, as the F/B increases, the number of powder particles in the mortar rises, thereby increasing the internal friction points within the mortar. This enhances the frictional and obstructive interactions between particles when the mortar is subjected to external forces, resulting in macroscopic increase in the average friction [34]. Secondly, with the increase in the F/B, the structure of the mortar becomes more compact. This denser structure exhibits greater resistance to deformation under external forces, leading to an increase in the average friction.

Furthermore, by comparing the influence of temperature on the average friction at the same F/B, it was observed that the average friction of the rubber asphalt mortar at −20 °C and −10 °C is generally comparable. However, the standard deviation of the average friction at −20 °C is consistently smaller than that at −10 °C, with absolute deviations of 1.0%, 2.5%, 1.9%, and 4.3%, respectively. Moreover, as the F/B increases, the difference in standard deviation becomes more pronounced, indicating that the fluctuation amplitude of the friction curves becomes more significant at higher F/B. This phenomenon may be attributed to the decrease in the glass transition temperature of the rubber asphalt mortar with higher F/B [36]. As a result, at −10 °C, the molecular motion within the rubber asphalt mortar remains relatively active, leading to greater variability in the friction between the rubber asphalt mortar and the friction pair materials at −10 °C for different F/B.

As shown in Figure 9b, at −20 °C, the average DFC of rubber asphalt mortar with F/B of 0.8, 1.0, and 1.2 increased by 6.25%, 14.7%, and 20.1%, respectively, compared to that with F/B of 0.6. At −10 °C, the increases were 6.8%, 15.7%, and 21.7%, respectively. This indicates that, within the range of commonly tested F/B in this study, the average DFC of rubber asphalt mortar increases with the rise in the F/B. Additionally, by analyzing the influence of temperature on the average DFC under the same F/B, it was found that the average DFC at −10 °C was consistently higher than that at −20 °C. This phenomenon may be attributed to reasons similar to those discussed earlier regarding the effect of temperature on the average DFC of rubber asphalt mortar with different powder contents. Furthermore, it was observed that, at the same F/B, the average DFC at −10 °C increased by 0.6%, 1.3%, 1.6%, and 2.1%, respectively, compared to that at −20 °C. The larger the F/B, the greater the difference in the average DFC between the two temperatures. This further confirms that, within the experimental range of this study (F/B of 0.6–1.2), higher F/B resulting in greater adhesive friction between the rubber asphalt mortar and the friction pair materials. It should be noted that the commonly used calcium carbonate filler was adopted to prepare asphalt mortar in this study. Given the performance differences between calcium carbonate filler and other fillers (e.g., hydrated lime and cement), the research results are only applicable to the calcium carbonate filler system, and the applicability of the research conclusions to asphalt mortar systems incorporating the aforementioned alternative fillers requires further verification.

### 3.4. Influence of Aging on the Frictional Properties of Rubber Asphalt Mortar

**Figure 10:**
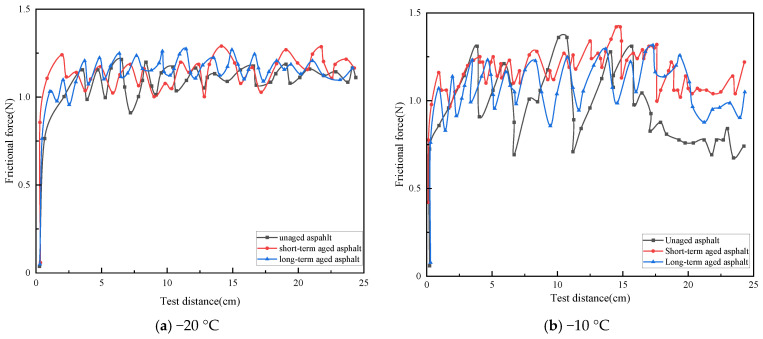
Friction force–distance curves of rubber asphalt mortar at different aging levels.

The rubber asphalt mortar, which was prepared with medium-particle-sized mineral filler, filler-to-binder ratio (F/B) of 0.6, and rubber powder content of 20%, was used to study and analyze the influence of different aging degrees on the adhesion and friction of the rubber asphalt mortar. The curve of the friction force of the rubber asphalt mortar versus the test distance is shown in Figure 10.

**Figure 11:**
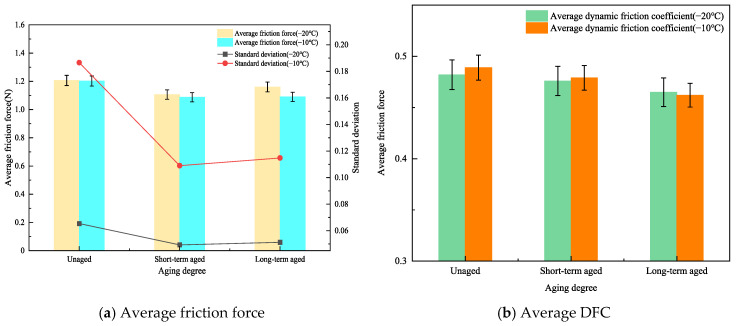
Average friction force and DFC of rubberized asphalt mortar with different aging levels.

As shown in Figure 10a, at −20 °C, the friction force curves of rubber asphalt mortar with different degrees of aging have similar fluctuation characteristics, with a fluctuation range of approximately 0.9 to 1.2 N. At −10 °C, all three curves fluctuate around 1.0 N on the *y*-axis. Among them, the original asphalt friction force curve has the largest fluctuation amplitude, followed by that of the long-term aged curve, and the short-term aged curve has the smallest amplitude. The average friction force and DFC of rubber asphalt mortar under different aging degrees are calculated and shown in Figure 11.

As shown in Figure 11a, at −20 °C, the original rubber asphalt mortar has the highest average friction force, which is 3.7% and 8.3% higher than those of long-term and short-term aged rubber asphalt mortar, respectively. At −10 °C, similar trend is observed, with the long-term and short-term aged rubber asphalt mortar having average friction forces 9.3% and 9.5% lower than the original mortar. It is hypothesized that increased aging degree can induce the transformation of crumb rubber surface morphology from porous structure to smooth and dense, and this morphological change may simultaneously reduce the elasticity and increase the rigidity of the mortar [37]. As a result, when subjected to external forces, its deformation capacity weakens, altering its contact and interaction with the rubber counterpart and thereby reducing the friction force.

In addition, the higher the aging degree, the smaller the standard deviation of the average friction force of the rubber asphalt mortar. At −20 °C, the standard deviation of the average friction force is smaller than that at −10 °C, and the difference in standard deviation decreases with increasing aging degree. This phenomenon is primarily attributed to the lower glass transition temperature associated with higher aging degrees [38]. At −20 °C, the molecular chain movement of the rubber asphalt mortar is inhibited, resulting in relatively fixed internal state and smaller standard deviation. At −10 °C, the molecular chains have some mobility, making the material properties more affected by temperature fluctuations and other factors. This increased mobility consequently leads to greater performance disparities within the material during friction, thereby increasing the standard deviation.

It can be seen from Figure 11b that the average DFC of the rubber asphalt mortar decreases as the degree of aging intensifies. At −20 °C, the average DFC of the rubber asphalt mortar during short-term aging and long-term aging is 1.2% and 3.5% lower, respectively, than that of the virgin asphalt. At −10 °C, they are 2.1% and 5.5% lower, respectively, than the average DFC of the virgin asphalt. As the degree of aging intensifies, the rubber asphalt mortar undergoes oxidative cross-linking reactions, which in turn elevates its glass transition temperature and simultaneously reduces the viscoelastic compliance of the mortar [39]. During the interaction process at the friction interface, the decrease in compliance may suppress the hysteretic energy dissipation effect between the mortar and the surface of the friction pair, thereby leading to a reduction in the average dynamic friction coefficient (DFC).

Meanwhile, when observing the influence of different temperatures on the average DFC under the same degree of aging, compared with −20 °C, the average DFC at −10 °C increases by 1.4%, 0.6%, and −0.6%, respectively, without showing obvious differences. This indicates that temperature exerts no significant effect on the frictional properties of rubber asphalt mortar with different aging degrees under negative temperature conditions.

### 3.5. Significance Analysis of Friction Properties Factors for Rubber Asphalt Mortar

The significant effects of temperature, crumb rubber content, mineral powder particle size, mineral filler-to-bitumen ratio (F/B), and aging degree on the dynamic friction coefficient (DFC) of rubberized asphalt mortar were systematically evaluated through multiple regression analysis and one-way ANOVA, with the results quantitatively summarized and statistically analyzed in Table 8 (*p* < 0.05).

**Figure 12:**
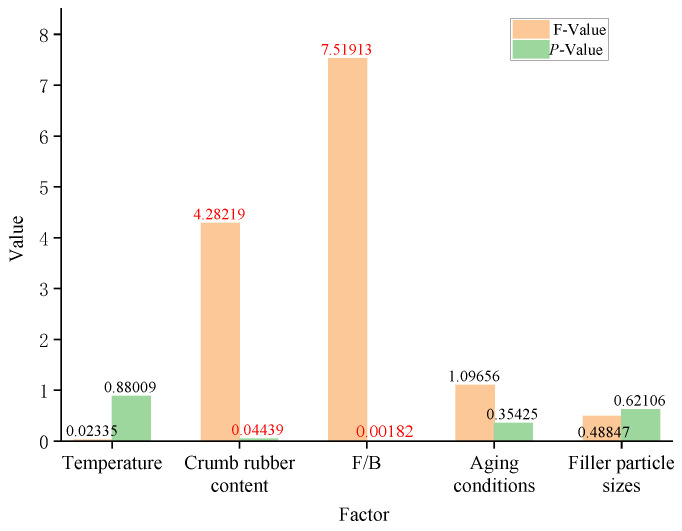
Influence of various factors on the frictional properties of rubberized asphalt mortar (ANOVA analysis).

As shown in Table 8 and Figure 12, the filler-to-binder ratio and crumb rubber content are identified as significant factors affecting the DFC of rubberized asphalt mortar (*p* < 0.05). Specifically, the filler-to-binder ratio is an extremely significant influencing factor (*p* = 0.02), and the crumb rubber content exerted a marginally significant effect on the friction properties (*p* = 0.044387, which did not meet the significance criterion after correction). In contrast, the filler particle size, aging degree, and temperature have no statistically significant impact on the DFC of rubberized asphalt mortar (the *p*-values of all these variables are >0.1).

However, it should be further clarified that the reason for the insensitivity to the mineral powder particle size may lie in the limited variation in the particle size of the test samples; the range of filler particle sizes is narrow, only from 0 mm to 0.075 mm, which fails to cause measurable changes in the surface roughness parameters. Although theoretical analysis indicates that the effect of microtexture on the friction coefficient in this experiment is negligible, this factor cannot be completely ruled out in practical applications.

There are differences between the rubber block adopted in this study and the complex formulation of tire treads in practical engineering, so the measured friction coefficient may deviate from the actual friction characteristics between pavement and tires. In addition, the experiment did not consider the effects of actual service conditions such as water film adhesion and contaminant coverage (e.g., oil stains and dust). Therefore, the research conclusions are only applicable to dry winter conditions and cannot be directly extended to humid or contaminated environments. It is necessary to further study different mineral powder particle sizes that can change the surface roughness of rubberized asphalt.

## 4. Conclusions

In order to clarify the relationship between the material composition and parameter changes in rubber asphalt mortar and its friction performance, a friction coefficient tester was used to analyze the friction force and friction coefficient of rubber asphalt mortar with different rubber powder contents, mineral powder particle size, powder–binder ratio, and aging degree under negative temperature. The following conclusions were obtained.

(1) Studies have shown that the frictional performance of rubberized asphalt mortar under negative temperature is comprehensively influenced by multiple factors, including crumb rubber content, filler-to-binder ratio (F/B), mineral filler particle size, and aging conditions. The F/B was identified as a significant influencing factor on the friction properties of rubber asphalt mortar (*p* < 0.01, after correction); the crumb rubber content exerted a marginally significant effect on the friction properties (*p* = 0.044387, which did not meet the significance criterion after correction).
(2) Increasing rubber powder content (10–30 wt%) significantly improved both friction force and DFC. Higher rubber content (30%) decreased temperature susceptibility. Lower temperatures (−20 °C) reduced friction force fluctuations (SD: 0.05–0.08 vs. 0.10–0.13 at −10 °C). The friction performance of rubber asphalt mortar increased with the F/B. Higher F/B exacerbated temperature-induced DFC variations, with F/B = 1.2 showing 2.1% DFC increase from −20 °C to −10 °C versus 0.6% for F/B = 0.6.
(3) At −10 °C and −20 °C, coarse, medium and fine mineral fillers exhibited negligible differences in average friction force (Δ < 2.7%), Reduced friction force fluctuations at −20 °C (SD ≈ 0.06) versus −10 °C (SD = 0.12–0.18). Both short-term and long-term aging reduced friction performance, with long-term aged mortar showing 8.3% lower friction force at −20 °C and 5.5% lower DFC at −10 °C versus virgin materials. Aged mortar exhibited narrower friction force standard deviations (SD reduced by 2.1–4.3% at −20 °C).

This study analyzed the effects of rubber asphalt mortar parameters on its frictional performance, which can provide data support and a theoretical basis for further understanding and enriching tire–pavement frictional performance. Our next research work will focus on (1) expanding the mineral filler particle size range and using real tire materials, (2) quantifying Tg of rubberized asphalt mortar, verifying its correlation with friction fluctuation, and (3) the relationship between the microscopic morphology and friction performance of asphalt mortar.

## Figures and Tables

**Table 1:** Parameters of the sample.

Crumb Rubber Content (%)	Mineral Powder Particle Size	Filler-to-Binder Ratio	Aging Conditions
0	medium (≤0.075 mm)	1.0	unaged asphalt
10	medium (≤0.075 mm)	1.0	unaged asphalt
20	medium (≤0.075 mm)	1.0	unaged asphalt
30	medium (≤0.075 mm)	1.0	unaged asphalt
20	coarse (0.053–0.075 mm)	1.0	unaged asphalt
20	fine (≤0.053 mm)	1.0	unaged asphalt
20	medium (≤0.075 mm)	0.6	unaged asphalt
20	medium (≤0.075 mm)	0.8	unaged asphalt
20	medium (≤0.075 mm)	1.2	unaged asphalt
20	medium (≤0.075 mm)	0.6	short-term aged asphalt
20	medium (≤0.075 mm)	0.6	long-term aged asphalt

**Table 2:** Properties index of 70# asphalt.

Items		Units	Specification Values	Measured Values
25 °C penetration/(100 g, 5 s)		0.1 mm	60~80	67.6
Softening point/(R&B)		°C	≥46	47.0
10 °C ductility		cm	≥20	25.2
RTFOT (163 °C, 85 min)	Mass loss	%	≤±0.8	−0.110
	Penetration ratio	%	≥65	69
	Ductility/10 °C	cm	≥6	6.0

**Table 3:** Properties index of rubber powder.

Items	Units	Specification Values	Measured Values
Density	g/cm^3^	1.1~1.2	1.12
Moisture content	%	<1	0.55
Iron content		<0.01	0.009
Fiber content		<0.5	0.07
Ash content		≤8	7.28
Acetone extract		6~16	7.26
Carbon black content		≥28	31
Rubber hydrocarbon content		42~65	55

**Table 4:** Physical properties index of extracted oil.

Items	Aromatics Content	Flash Point	Viscosity	Density	Color	State
Units	%	°C	Pa·s (60 °C)	g/cm^3^		
Result	88	220	0.05	1.03	dark brown	Viscous

**Table 5:** Physical properties index of asphalt stabilizer.

Items	Melting Point	Flash Point	Density	Color	State
Units	°C	°C	g/cm^3^		
Result	114	168	2.36	Yellow	Powder

**Table 6:** Properties index of mineral filler.

Items		Units	Requirement	Result
Apparent density		g·cm^−3^	≥2.50	2.725
Moisture content		%	≤1.0	0.42
Hydrophilic coefficient		-	<1	0.60
Size range	<0.6 mm	%	100	100
	<0.15 mm	%	90–100	98
	<0.075 mm	%	75–100	93.2

**Table 7:** Test Parameters of Friction Coefficient Tester.

Test Parameters	Weight of Weight	Sliding Speed of Slider	Travel Distance of Slider	Test Force Accuracy	Hardness of Rubber Block	Composition of Rubber Block	Specimen Size	
							Rubber Block	Test Plate
Technical Requirements	200 ± 2 g	100 ± 10 mm/min	25 cm	±0.02 N	75 HA	SBR/NR (7:3) + Carbon Black: 40 phr	63 × 63 mm	80 × 300 mm

**Table 8:** Results of the significance analysis.

Factor	SS	df	MS	F	*p*-Value	F Crit
Temperature	2.2 × 10^−5^	1	2.2 × 10^−5^	0.023348	0.880087	4.351244
Crumb rubber content	0.005736	3	0.001912	4.282195	0.044387	4.066181
F/B	0.010494	3	0.003498	7.519135	0.001823	3.159908
Aging conditions	0.001952	2	0.000976	1.09656	0.354248	3.521893
Filler particle sizes	0.000923	2	0.000461	0.488467	0.621063	3.521893

## Data Availability

The original contributions presented in this study are included in the article. Further inquiries can be directed to the corresponding authors.
